# Comparative effects of 17% ethylenediaminetetraacetic acid and 9% etidronic acid applied with different irrigant activation techniques on the release of growth factors from dentin: in vitro study

**DOI:** 10.1186/s12903-024-04336-0

**Published:** 2024-05-27

**Authors:** Arzu Kaya Mumcu, Safa Kurnaz, Gülsen Kiraz, Meliha Koldemir Gündüz

**Affiliations:** 1 Department of Endodontics, Faculty of Dentistry, Kutahya Health Sciences University, Kutahya, Türkiye; 2 Department of Basic Sciences of Engineering, Faculty of Engineering and Natural Sciences, Kutahya Health Sciences University, Kutahya, Türkiye

**Keywords:** EDDY, Ethylenediaminetetraacetic acid, Etidronic acid, Growth factors, XP-endo Finisher

## Abstract

**Background:**

Growth factors embedded in the extracellular matrix of the dentin play an important role in the migration, proliferation, and differentiation of dental pulp stem cells in regenerative endodontics. In regenerative endodontic treatments, the type of irrigation solution used is crucial for the release of growth factors (GFs) from the dentin matrix. This study evaluated the effectiveness of different irrigant activation techniques (IAT) using two different chelating agents, 17% ethylenediaminetetraacetic acid **(**EDTA) and 9% etidronic acid (HEDP), in terms of their GF release.

**Methods:**

Seventy-two mandibular premolar teeth were prepared to simulate an open apex. The root fragments were irrigated with 20 ml of 1.5% sodium hypochlorite and 20 ml of saline solution. Eight root fragments were randomly separated for the control group, and the remaining 64 fragments were randomly separated into eight groups based on two different chelating agents (17% EDTA and 9% HEDP) and four different IAT ((conventional needle irrigation (CNI), passive ultrasonic irrigation (PUI), sonic activation with EDDY, and XP-endo Finisher (XPF)). TGF-β1, VEGF-A, BMP-7 and IGF-1 release levels were determined using an ELISA, and statistical analysis was performed using the Kolmogorov–Smirnov test, ANOVA, and the Tukey test (*p* < .05).

**Results:**

Compared to the control group, the experimental groups showed significantly higher GF release when using EDTA or HEDP. Among the activation groups, the EDDY group triggered the highest GF release, and the CNI group triggered the lowest.

**Conclusions:**

IAT with EDTA and HEDP can increase GF release, with EDDY being the most effective IAT method. Using chelating agents with IAT may be beneficial in regenerative endodontic treatments.

## Background

Regenerative endodontic procedures (REPs) aim to prevent or heal apical periodontitis and ensure the continued development of roots by stimulating the regeneration of the pulp-dentin complex [1]. REPs, various studies have shown, facilitate root development in immature permanent teeth [2, 3]. They help recover vitality and promote apical healing even in mature necrotic teeth [4]. To successfully regenerate the pulp-dentin complex, stem cells, growth factors (GFs), and scaffolding are essential. However, the literature on GFs, unlike that of stem cells and scaffolds, is limited [5, 6].

The GFs embedded in the extracellular matrix of dentin are critical for the migration, proliferation, and differentiation of dental pulp stem cells (DPSCs) [7, 8]. The factors that induce differentiation to DPSCs include the transforming growth factor-β (TGF-β), vascular endothelial growth factors (VEGFs), bone morphogenetic proteins (BMPs), insulin-like growth factors (IGFs), fibroblast growth factors (FGFs), platelet-derived growth factors (PDGFs), epidermal growth factors (EGFs), and interleukins [9]. Applying dental materials, acids, or chelating agents can demineralize the dentin matrix, which can then solubilize and expose the matrix-bound GFs [10].

Chelating agents used to demineralize the dentin matrix can induce the release of GFs. These released GFs are known to influence cellular responses. Irrigants can influence stem cell proliferation and migration by altering dentin structure. In this context, the type of irrigation solution used becomes paramount for optimizing GF release [11]. Ethylenediaminetetraacetic acid (EDTA) irrigation is a recommended final step in REPs. It serves the dual purpose of releasing GFs from the dentin matrix and eliminate the negative effects of sodium hypochlorite (NaOCl) [12]. Studies have demonstrated that conditioning root canal dentin with EDTA promotes cell survival, attachment, and differentiation of stem cells, likely due to the associated release of GFs [26]. Etidronic acid (HEDP) is a non-toxic, weakly acidic bisphosphonate that is less aggressive on root dentine while removing the smear layer. HEDP is also used in endodontics due to its chelating properties, which do not adversely affect NaOCl [13]. However, its effect on GF release from the dentin matrix remains understudied [14].

With root canal irrigation, dentin tubules and collagen fibrils can be exposed and made more suitable for stem cell adhesion. Since conventional needle irrigation (CNI) ineffectively delivers chelating agents to the intricate regions of root canals, many studies have investigated the use of irrigant activation techniques (IAT). They investigated the efficacy of passive ultrasonic irrigation (PUI), EDDY sonic activation (VDW, Munich, Germany), and XP-endo Finisher (XPF; FKG, La Chaux-de-Fonds, Switzerland) in removing debris and smear layer, and antibacterial activity. Although studies have proven the effectiveness of IAT in increasing canal disinfection [15–17], their effectiveness in triggering GF release remains underexplored [18–20].

This investigation aimed to elucidate the effects of various IATs on the release of four GFs (TGF-β1, VEGF-A, BMP-7, and IGF-1) from the dentin matrix. The investigation utilized two chelating agents, EDTA and HEDP, in conjunction with four distinct IATs: CNI, PUI, EDDY, and XPF. The null hypothesis was that the different IAT and chelating agents were not different in their effectiveness in terms of GF release.

## Methods

The Ethics Committee of Kutahya Health Sciences University (no: 2022/05–21) approved the study protocol. The sample size was calculated at the effect size of 0.5, type 1 error 0.05, and power of 0.83 using G* Power (ver.3.1.9.7) [19]. Each tooth underwent a meticulous examination under a dental operating microscope (OMS 2350, Zumax Company, China) to identify and exclude any specimens exhibiting caries, cracks, or fractures. Periapical radiographs were obtained in both buccolingual and mesiodistal directions to confirm the absence of internal resorption or calcification, and to verify the presence of a single root canal. Seventy-two extracted human teeth satisfying the selection criteria—mandibular premolar with a single root and teeth without caries, fractures, calcifications, resorptions, and anatomic aberrations— were selected. Samples were selected from teeth extracted for orthodontic or periodontal reasons with informed consent in the Department of Oral and Maxillofacial Surgery, Faculty of Dentistry, Kutahya Health Sciences University.

### Study procedures

The teeth were flushed with phosphate-buffered saline (PBS, pH: 7.4) (Biomatik, Ontario, Canada), the periodontal tissues were removed, and the teeth were kept in 0.1% thymol solution at 4 °C until their use. The teeth were decoronated with sterile diamond burs (Diatech; Coltene Whaledent, Altstatten, Switzerland) under water cooling to create a standardized canal length of 12 ± 1 mm from the apex. To establish standardized open apices with a targeted diameter of 1.1 mm, the root fragments were sequentially prepared using Gates Glidden drills #1, #2, #3, and #4 (Dentsply Sirona, Ballaigues, Switzerland). A size #15 K-file was advanced within the root canal under the dental operating microscope until the file tip reached the prepared open apex. Subsequently, a silicone stopper was positioned at the reference point. The distance between the base of the stopper and the tip of the K-file was then measured. The root fragments were covered with nail polish, leaving only the inner root canal dentin surface uncovered. The composite resin (3 M ESPE, St. Paul, MN, USA) was applied to the apical root fragments to perform irrigant activation techniques in a closed system in order to generate turbulences and a hydrodynamic flow within the irrigant. The root fragments were then irrigated with 1.5% NaOCl (Wizard, Rehber Chemistry, Istanbul, Turkey) (20 mL, 5 min) and saline (20 mL, 5 min). Eight fragments were randomly separated for the control group; the remaining 64 were placed in a microcentrifuge tube (Nest Biotechnology, Wuxi, China) with a silicone-based impression material (Zetaplus, Zhermack, Rome, Italy).

The irrigation solutions used were 1.5% NaOCl, 17% EDTA (Promida, Eskişehir, Turkey), and 9% HEDP (Bostonchem, Boston, USA). The HEDP solution (60% aqueous, CAS: 2809-21-4) was diluted with ultrapure water to achieve a concentration of 9% (weight/volume). The resulting irrigation solution was stored in a glass bottle at room temperature before experimental use.

### Study groups

After chemo-mechanical preparation, sixty-four root fragments were randomly separated into eight groups (*n* = 8) based on the final irrigation procedure (two different chelating agents and four different IATs) (Table 1).

**Table 1:** Experimental groups and irrigation protocols

Irrigant Activation Technique	Groups (*n* = 8)	Irrigation Protocols (20mL/5min)
**-**	**Control**	1.5% NaOCl + Saline
**CNI**	**CNI-E**	1.5% NaOCl + Saline + 17% EDTA
	**CNI-H**	1.5% NaOCl + Saline + 9% HEDP
**PUI**	**PUI-E**	1.5% NaOCl + Saline + 17% EDTA
	**PUI-H**	1.5% NaOCl + Saline + 9% HEDP
**XPF**	**XPF-E**	1.5% NaOCl + Saline + 17% EDTA
	**XPF-H**	1.5% NaOCl + Saline + 9% HEDP
**EDDY**	**EDDY-E**	1.5% NaOCl + Saline + 17% EDTA
	**EDDY-H**	1.5% NaOCl + Saline + 9% HEDP

*CNI, conventional needle irrigation; PUI, passive ultrasonic irrigation; XPF, XP-Endo Finisher; E, EDTA; H, HEDP.

#### Control group

In the control group, 20 mL of 1.5% NaOCl (5 min), followed by 20 mL saline (5 min), was used for irrigation.

#### Experimental groups

Group CNI-E and Group CNI-H: The 30-gauge side-vented irrigation needle (Fanta Dental, Istanbul, Turkey) was placed 1 mm short of the working length (WL), and 20 ml of 17% EDTA or 9% HEDP solutions were applied for 5 min with an up-and-down movement of 4–5 mm in the root canal. In the CNI group, the volume of irrigation solutions and irrigation time were similar to those in the activation groups. The flow rate was approximately 4 mL min^− 1^.

Group PUI-E and Group PUI-H: An Irri S 21/25 ultrasonic tip (VDW, Munich, Germany) was coupled to the VDW Ultra ultrasonic (VDW, Munich, Germany), and the power was set to 30. The root fragments were filled with 2 mL of 17% EDTA or 9% HEDP solution and activated for 30 s. The ultrasonic tip was inserted 2 mm short of the WL. Subsequently, the root fragments were refilled with fresh 2 mL of 17% EDTA or 9% HEDP solution and activated for an additional 30 s. This process was repeated until a total of 20 mL of irrigation solution had been applied over a 5-minute period.

Group XPF-E and Group XPF-H: The XPF (size 25, 0.00 taper) was first immersed in 35 °C water to ensure the phase transformation and then placed in the root canal. The instrument was coupled to a VDW Gold motor (VDW, Munich, Germany) in a rotating motion at 800 rpm and 1.0 Ncm torque according to the manufacturer’s instructions. The root fragments were filled with 2 mL of 17% EDTA or 9% HEDP solution, and the XPF was inserted 1 mm short of the WL and activated for 30 s. Activation was performed with gentle and slow up-and-down movements of 7–8 mm. Subsequently, the root fragments were refilled with fresh 2 mL of 17% EDTA or 9% HEDP solution and activated for an additional 30 s. This process was repeated until a total of 20 mL of irrigation solution had been applied over a 5-minute period.

Group EDDY-E and Group EDDY-H: The EDDY instrument was coupled to the Micron TA 200 (Micron, Tokyo, Japan). The root fragments were filled with 2 mL of 17% EDTA or 9% HEDP solution, and the EDDY tip was inserted 2 mm short of the WL and activated for 30 s. A pecking motion was used for activation. Subsequently, the root fragments were refilled with fresh 2 mL of 17% EDTA or 9% HEDP solution and activated for an additional 30 s. This process was repeated until a total of 20 mL of irrigation solution had been applied over a 5-minute period.

### Sample collection and enzyme-linked immunosorbent assay (ELISA)

The root fragments were irrigated using 20 mL saline for 5 min. Each fragment was then placed into a microcentrifuge tube containing 1 mL PBS (pH: 7.4) and kept at 37 °C for 24 h. The TGF-β1, VEGF-A, BMP-7, and IGF-1, which were released into the medium because of irrigation, were determined using an ELISA kit (Bostonchem, Boston, USA). The root canal volume (RCV) (mm^3^) and the volume of the medium used in the ELISA (at least 0.1 mL/ = 100 mm^3^) had a significant difference. The actual GF concentration was then calculated by following previous studies [8, 19]. RCV (V_(Canal)_) was calculated using the following formula from the cone beam computed tomography (CBCT) (Instrumentarium OP300, Tuusula, Finland) image. The expressions in the formula are L; root length (mm), D; coronal diameter (mm), and d; apical diameter (mm).

$${V_{(Canal)}} = {\pi}L\left\{ {{{\left( {D/2} \right)}^2} + \left( {D/2} \right)\left( {d/2} \right) + {{\left( {d/2} \right)}^2}} \right\}/3$$

After measuring the RCV, the final concentration of GFs (C_canal_) was calculated using the following formula:

$${C_{(Canal)}} = {C_{(Elisa)}} \times {V_{(Collecting{\text{ }}Medium)}}/{V_{(Canal)}}$$

### Statistical analysis

SPSS (version 21.0; IBM, Armonk, NY, USA) was used for statistical analysis. After verifying data normality using the Kolmogorov–Smirnov test, data was analyzed using analysis of variance (one-way ANOVA), followed by Tukey’s test. The mean results of the activation protocols of the EDTA and HEDP groups were compared using an independent t-test. The data, presented as mean standard deviation, were considered significant at *P* < .05.

## Results

**Fig. 1:**
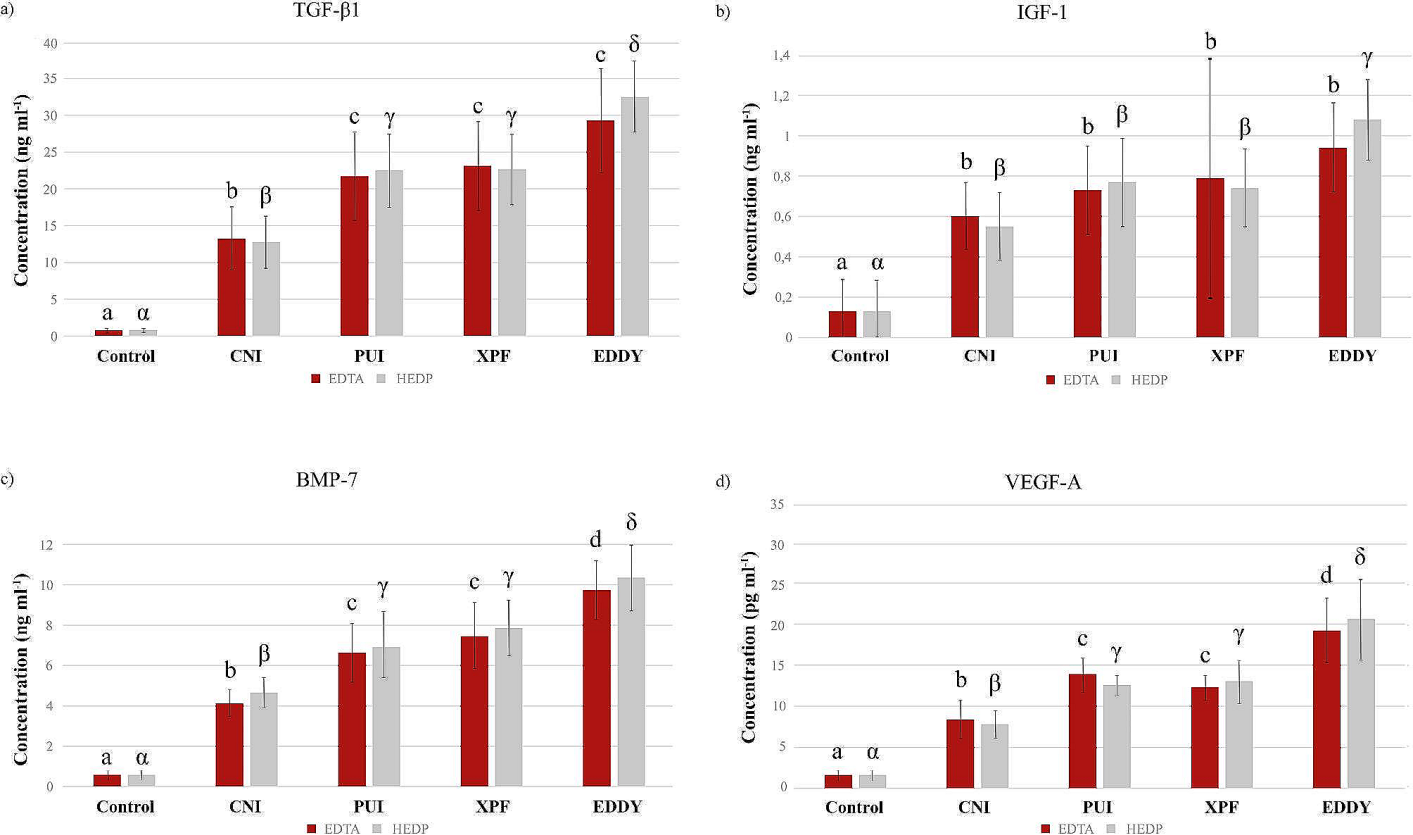
The GF release levels from root segments treated with chelating agents and IAT at 24 h. * Groups with statistical differences are indicated with different letters

The experimental groups triggered significantly higher GF release than the control group (*P* < .05): the EDDY group triggered the greatest GF release, while the CNI group triggered the lowest. There was no statistically significant difference in the GF release between the EDTA and HEDP groups, regardless of the IAT (*P* > .05) (Table 2; Fig. 1).

**Table 2:** The mean and standard deviations of growth factor release levels with EDTA and HEDP

		Control	CNI	PUI	XPF	EDDY
**TGF-β1** (ng/ml)	**17% EDTA**	0,82 ± 0,27^a^	13,21 ± 4,8^b^	21,71 ± 6,63^c^	23,13 ± 6,20^c^	29,31 ± 6,95^c^
	**9% HEDP**	0,82 ± 0,27 ^α^	12,76 ± 3,81^β^	22,52 ± 4,95 ^γ^	22,66 ± 5,42 ^γ^	32,49 ± 5,34 ^δ^
**IGF-1** (ng/ml)	**17% EDTA**	0,13 ± 0,15^a^	0,60 ± 0,14^b^	0,73 ± 0,22^b^	0,79 ± 0,59^b^	0,94 ± 0,24^b^
	**9% HEDP**	0,13 ± 0,15 ^α^	0,55 ± 0,13 ^β^	0,77 ± 0,23 ^ß^	0,74 ± 0,18 ^ß^	1,08 ± 0,21 ^γ^
**BMP-7** (ng/ml)	**17% EDTA**	0,59 ± 0,13^a^	4,13 ± 1,03^b^	6,63 ± 1,65^c^	7,45 ± 1,83^c^	9,73 ± 1,60^d^
	**9% HEDP**	0,59 ± 0,13 ^α^	4,66 ± 1,34 ^β^	6,91 ± 1,69 ^γ^	7,86 ± 1,52 ^γ^	10,36 ± 1,81 ^δ^
**VEGF-A** (pg/ml)	**17% EDTA**	1,66 ± 0,46^a^	8,39 ± 2,26^b^	13,91 ± 1,79^c^	12,30 ± 1,74^c^	19,22 ± 3,5^d^
	**9% HEDP**	1,66 ± 0,46 ^α^	7,82 ± 1,58 ^β^	12,60 ± 2,20 ^γ^	13,06 ± 2,35 ^γ^	20,63 ± 5,1 ^δ^

*Different Latin alphabetical letters at lines indicate a significant difference for EDTA groups (*p* < .05)

*Different Greek alphabetical letters at lines indicate a significant difference for HEDP groups (*p* < .05)

The release of TGF-β1 (ng/ml) and IGF-1 (ng/ml) was evaluated with different IAT in the EDTA and HEDP groups. The study found a statistically significant difference between the activation groups and the CNI groups (*P* < .05). In the EDTA groups, no significant difference was found between the activation groups (*P* > .05), In the HEDP groups, while the EDDY group was found to be statistically significantly higher than all groups (*P* < .05), there was no significant difference between the PUI and XPF groups (Fig. 1a and b) (*P* > .05).

The release of BMP-7 (ng/ml) and VEGF-A (pg/ml) was evaluated with different IAT in the EDTA and HEDP groups. The study found a statistically significant difference between the activation groups and the CNI groups (*P* < .05). Despite no significant difference between the PUI and XPF groups, the EDDY group was found to be statistically significantly higher than all groups (Fig. 1c and d) (*P* < .05).

## Discussion

This study investigated the influence of different chelating agents and IAT on the release of four GFs from the dentin matrix of extracted premolar teeth. It found that, while different chelating agents did not significantly affect GF release, IAT significantly increased it. The null hypothesis was partially accepted.

Previous investigations on GF release from the dentin matrix using different chelating agents or IAT [6, 8, 21] employed different methods—such as dentin powder or dentin disk—that have limitations: dentin powder might maximize GF release, and dentin disks do not reflect clinical application due to growth factor release from the entire coronal disk surface [9, 11, 14, 21]. Furthermore, the in vivo scenario necessitates consideration of two key factors influencing GF release. Firstly, growth factors are released exclusively from the inner dentin surface of the root. Secondly, the volume of the root segment directly impacts the level of released GF. Recognizing these limitations, the present study adopted the use of standardized root fragments. This approach aligns with the in vivo setting, as GF release originates from the inner dentin surfaces of the root [8, 19]. Additionally, to minimize confounding factors, the root fragments were standardized in length, and their outer surfaces were coated with nail polish [19].

Previous studies immersed dentin disks in EDTA to determine the GF level [9, 11]. In clinical settings, EDTA is removed after irrigation to ensure that the released GFs are important for tissue regeneration because apical bleeding—necessary for tissue regeneration—is induced after EDTA removal [8]. To represent the clinical setting, this study immersed the root fragments in PBS after irrigation with EDTA or HEDP. The GF release was measured after 24 h [19].

RCV is smaller than the volume used for ELISA, which is a limitation of previous studies that used the volume of solution collected for ELISA to calculate the concentrations of GFs, rather than the RCV space [8, 9, 11]. To better approximate the clinical setting, this study calculated RCV using CBCT and determined the actual GF concentration based on each fragment’s RCV [8, 19].

TGF-β1, VEGF-A, BMP-7, and IGF-1 are important regulators of angiogenesis, odontoblast differentiation, and hard tissue formation [19]. TGF-β1 is an important molecule that mediates the signaling of odontoblast differentiation and mineralization and exerts a chemotactic effect on DPSCs [11, 22]. VEGF-A, a potent angiogenic factor, promotes the proliferation and migration of endothelial cells [23]. BMP-7 stimulates stem cell differentiation and induces dentin formation [24]. IGF-1 induces human DPSC proliferation and osteo/odontogenic differentiation and provides mineralization and cell differentiation [25]. Due to their important effects on regeneration, this study investigated TGF-β1, VEGF-A, BMP-7, and IGF-1 from the root fragments.

Chelating agents’ demineralization of the dentin matrix can induce the release of the above GFs, which can elicit cellular responses even at low concentrations [26]. The effect may vary based on solution type and concentration, and the irrigants can directly affect cells or secrete signal molecules to induce migration and differentiation [7]. EDTA is recommended for the final irrigation in regenerative treatment to release GFs from the dentin matrix and prevent the adverse effects of NaOCl [27]. The EDTA conditioning of root canal dentin supports the cell survival, attachment, and differentiation of DPSCs and GF release from the dentin matrix [26, 28]. Various chemical substances, such as phosphoric acid, citric acid, phytic acid, and maleic acid, have been tested for their effectiveness in releasing GFs in regenerative endodontics [6, 14, 19, 29]. Although HEDP has been recommended for clinical use due to its chelating properties, research on its use as a final irrigation agent to release GFs in regenerative endodontics is limited [13, 14]. This study evaluated the effectiveness of HEDP in terms of GF release by comparing it with that of EDTA.

The study found EDTA and HEDP to be equally effective in GF release, regardless of whether irrigation was activated or the type of IAT used. Sungur et al. [14] compared the effects of 17% EDTA, 9% HEDP and, 1% phytic acid on the release of TGF-β from dentinal disks and reported statistically insignificant differences between the groups—a finding consistent with that of this study. Another study found no significant difference in the smear layer removal capacity of 17% EDTA and 9% HEDP solutions—a finding corroborating EDTA and HEDP’s similar efficacy as irrigating solutions [30]. This similarity may be attributed to the solutions’ similar demineralization capabilities, which allow them to release GFs similarly.

REP success may be improved by increasing the disinfection of the dentin matrix using IAT, which can make dentin tubules and collagen fibrils suitable scaffolds for stem cell adhesion [19, 31]. Widbiller et al. [20], Aksel et al. [18], and Hançerlioğulları et al. [19] found that IAT application increased GF release from dentin. In the present study, GFs were released from both the EDTA and HEDP groups, and IAT increased the level of GF release unlike conventional syringe irrigation. Only a few studies have compared the release levels of TGF-β1, VEGF-A, BMP-7, and IGF-1 obtained with PUI with EDTA [18–20]. Moreover, no studies have compared its use with PUI using HEDP or investigated GF release levels using XPF and EDDY with EDTA and HEDP.

Studies have shown that XPF and PUI are more effective than needle irrigation in removing multispecies biofilm and eliminating intracanal bacteria and triple antibiotic paste from root canals. However, no statistically significant difference was found between the two groups [32–34]. This study also found no statistically significant difference in GF release between XPF and PUI, a finding that corroborates the similarities of both IAT. This study observed more GF release in the groups activated with EDDY. Compared to PUI and EDDY, as per past studies, EDDY has superior cleaning efficacy, dentin debridement, and endodontic sealer and intracanal medicament removal efficacy [34–36]. Additionally, based on comparisons of EDDY and XPF, EDDY can more effectively remove intracanal medicaments and endodontic sealers [34, 36]. This is because EDDY’s flexible soft polymer tip and its three-dimensional tip movement allows better penetration and activation of irrigation [35, 37]. The increased GF release this study found in the EDDY groups was maybe because of this as well.

The limitation of the present study was the evaluation of the GF levels in root fragments containing healthy dentin. However, the presence of biofilm may alter the amount of GF released, which may affect regenerative potential. Studies have shown that the presence of biofilm on the dentin surface reduces GF release [29, 38]. Many studies evaluating the effect of different chelation agents and IATs have measured the release of GFs from healthy dentin to simulate clinical conditions [6, 8, 14, 19, 20]. Therefore, healthy root dentin was used in this study. However, the amounts of GF release from healthy dentin may differ from in vivo cases.

The findings of this study revealed that IATs used with EDTA and HEDP could increase GF release, and EDDY activation was the most effective. According to the results obtained, HEDP may be an alternative solution to EDTA in the clinical application of REPs. Different IATs may have different clinical results in terms of the release of GFs. Therefore, future studies should evaluate the clinical impact of chelating agents applied with different irrigation activation methods on regenerative endodontic procedures.

## Conclusion

It was observed that both EDTA and HEDP chelating agents had similar effect on GF release and there was no statistically significant difference was observed between the efficacy of EDTA and HEDP solutions. Both chelating agents significantly increased GF release from the dentin matrix compared to the control group. EDDY activation was identified as the most effective method for stimulating GF release, regardless of the chelating agent used (EDTA or HEDP).

## Data Availability

The datasets for the current study are available from the corresponding author on reasonable request.

## Abbreviations

GFs: Growth factors
IAT: Irrigant activation techniques
EDTA: Ethylenediaminetetraacetic acid
HEDP: Etidronic acid
NaOCl: Sodium hypochlorite
CNI: Conventional needle irrigation
PUI: Passive ultrasonic irrigation
XPF: XP-endo Finisher
REPs: Regenerative endodontic procedures
DPSCs: Dental pulp stem cells
TGF-β: Transforming growth factor-β
VEGFs: Vascular endothelial growth factors
BMPs: Bone morphogenetic proteins
IGFs: Insulin-like growth factors
FGFs: Fibroblast growth factors
PDGFs: Platelet-derived growth factors
EGFs: Epidermal growth factors
PBS: Phosphate-buffered saline
RCV: Root canal volume
